# Unhealthy Food and Sugar-Sweetened Beverage Consumption Among Bangladeshi Elderly People and Their Sociodemographic Determinants: Findings From a Nationally Representative Cross-Sectional Study

**DOI:** 10.7759/cureus.61922

**Published:** 2024-06-07

**Authors:** Abu Ahmed Shamim, Fahmida Akter, Md. Mokbul Hossain, Farhana Rinky, Abu Abdullah Mohammad Hanif, Mehedi Hasan, Md. Showkat Ali Khan, Mohammad Aman Ullah, Md. Mafizul I Bulbul, Malay K Mridha

**Affiliations:** 1 Center for Non-communicable Diseases and Nutrition, BRAC James P Grant School of Public Health, BRAC University, Dhaka, BGD; 2 National Nutrition Services, Institute of Public Health Nutrition, Ministry of Health and Family Welfare, Dhaka, BGD

**Keywords:** geriatric nutrition, elderly, savory snacks, sugar, bangladesh, sugar-sweetened beverages, unhealthy snacks

## Abstract

Background: Savory crispy or fried snack (SCFS), sugary snack (SS), and sugar-sweetened beverage (SSB) consumption are associated with a higher prevalence of obesity and non-communicable diseases. So, we estimated the consumption of SCFS, SS, and SSB among elderly males and females in Bangladesh. We also reported the factors associated with their consumption using data from a nationwide cross-sectional study.

Methods: We interviewed 2,482 (51.52%) elderly males and 2,335 (48.47%) elderly females for the recall for the past seven days on the intake of SCFS, SS, and SSB from 82 randomly selected clusters from rural, non-slum urban, and slum areas in Bangladesh. Sociodemographic and anthropometry data were also collected.

Results: Consumption of SCFS, SS, and SSB for ≥1 time per week was reported by 884 (31.5%), 1,696 (66.1%), and 1,911 (69.3%) of the elderly males and 516 (20.1%), 1,367 (53.9%), and 1,171 (34.1%) of the elderly females, respectively. Both elderly males and females from slum and non-slum urban areas consumed more SSB than their rural counterparts. Higher level of television viewing was associated with increased frequency of intake of SCFS, SS, and SSB among elderly males and SSB intake among elderly females. Nutritional status was not associated with the consumption of these foods and drinks among the elderly; however, overweight males consumed SSB less frequently.

Conclusion: In Bangladesh, elderly males and females frequently consume unhealthy snacks and drinks. Considering their detrimental effect on health, it is necessary to reduce their consumption through policy and program measures and promote healthier foods and beverages.

## Introduction

The elderly stage of life constitutes an important phase of the human lifespan marked by distinct physiological and psychosocial characteristics. Like many other countries, the share of the elderly population is rapidly increasing in Bangladesh, and it is estimated that the number of elderly people in Bangladesh will reach 40 million in 2050 (20% of the total population) from 12.5 million (7.5%) in 2019 [1]. Contemporary developmental policies emphasize the importance of the health, nutrition, and overall well-being of the elderly to enhance their quality of life and societal benefits.

Consumption of a diverse and healthy diet plays an important role in protecting the health, well-being, and quality of life of the elderly [2]. However, it was reported earlier that consumption of an inadequately diversified diet (four or less food groups out of 10) is common [3], and the prevalence of both underweight and overweight is also high among the elderly population of Bangladesh [4]. Malnutrition among the elderly is a risk factor for increased morbidity and mortality as they may have suffered from chronic conditions that could be improved with proper nutrition; malnutrition in elderly people generally results from somatic causes such as problems in chewing or swallowing disorders, psychological problems such as depression, and socioeconomic problems such as lack of income, social deprivation, and loneliness, which calls for increase investment in elderly [5]. Among the factors associated with the deteriorating health and nutrition encountered by the elderly, the consumption of processed and nutritionally deficient foods and beverages has emerged as a prominent concern [6]. Processed foods in Bangladesh are typified by elevated concentrations of sodium, sugars, and saturated fats while lacking health-promoting constituents such as dietary fiber [7]; also, the edible oils used in Bangladesh for the preparation of processed foods are high in trans fats, which are likely to contribute to non-communicable disease risks [8]. These nutritional attributes render processed food consumption an important risk factor for a spectrum of adverse health outcomes among the elderly. A growing body of evidence is underscoring the association of increased consumption of processed and ultra-processed foods with the risk of diet-related non-communicable diseases such as hypertension [6], dyslipidemia [9], and frailty [10] among elderly people.

In cognizance of these growing concerns, several health-oriented organizations have formulated dietary guidelines intended to foster healthier eating practices among the elderly, with an emphasis on the importance of nutrient-dense foods and a reduction in the consumption of processed and nutritionally imbalanced alternatives.

As frequent intake of calorie-dense but nutrient-poor foods and drinks in late life may contribute to a higher prevalence of non-communicable disease and mortality, it is crucial to know how often these foods and drinks are consumed by the elderly, as well as the sociodemographic factors influencing their consumption. To our knowledge, no nationally representative research reported the consumption of savory and sweet foods and beverages among the elderly in Bangladesh.

Aim and objectives

The objective of this study is to shed light on the current void in academic research regarding the consumption of savory crispy or fried snacks (SCFS), sugary snacks (SS), and sugar-sweetened beverages (SSB) among Bangladeshi elderly and report the sociodemographic factors that influence their consumption using data sourced from a comprehensive nationally representative cross-sectional survey.

## Materials and methods

The Food Security and Nutrition Surveillance Project (FSNSP) has been implemented in Bangladesh since 1990 to collect data from women and children on a regular basis. In the 2018-2019 round of the FSNSP, elderly men and women were included for the first time in Bangladesh. The sample size was estimated to represent the findings for all administrative eight divisions of Bangladesh, and based on the prevalence of important variables, the estimated sample size for the elderly age group was 4,817. In rural areas, we randomly selected two districts from each of the eight divisions, followed by the random choice of a sub-district in each district. Two unions (a small administrative unit) were then randomly chosen from each sub-district. These unions were divided into 250-400 household segments, with two segments randomly selected from each union for enumeration. However, due to administrative and financial constraints, we collected data from 57 out of the planned 64 rural clusters. In urban areas, we collected data from 15 clusters instead of the intended 16, taking two clusters from each division. Additionally, we selected 10 slums, including two from major city corporations and one from each of the remaining six divisions. From each cluster, we interviewed 62 elderly individuals randomly selected from household lists. In total, 4,817 elderly individuals, consisting of 2,482 males and 2,335 females, were interviewed.

Quality control and data collection

Five data collection teams, each led by a supervisor and consisting of 4-5 data collectors, gathered information over a year, from October 6, 2018, to October 31, 2019. The training program for these teams, spanning five days, encompassed both fieldwork practices and classroom instruction and was conducted by the field coordinator and investigators. Each data collector had undergone anthropometric measurement standardization training according to procedures described in the standard reference [11]. The questionnaire was modified based on feedback and field-tested; about 5% of randomly selected interviews were re-conducted by the supervisor within 48 hours.

Outcome variables

The outcome variables and the methods of data collection are described previously in a paper exploring the consumption of these foods among adolescents from the same study sites [12]. In summary, we categorized three groups as unhealthy drinks and foods, regardless of their source according to the "optional categories" as described in the measurement of dietary diversity [13]. Savory crispy or fried snacks (SCFS) encompassed homemade or store-bought salty, spicy snacks such as pakoras, piaju, singaras, and samosas and commercial items such as chanachur and chips. Sugary snacks (SS) included South Asian milk-based dairy products, and desserts and snacks prepared with added sugar [14]. Sugar-sweetened beverages (SSB) are drinks with added sugars as defined by the CDC [15]. As part of a seven-day food frequency questionnaire, the intake of SCFS, SS, and SSB was measured by asking questions about each item with examples. For example, to collect data about the weekly frequency of SSB, the question was "In the last seven days, how many days did you drink SSB such as sweetened cold drinks, tea, juice, yogurt drinks, energy drinks, chocolate drinks, sweet malted drinks, Horlicks, etc.?" The interviewer recorded the number of days and, for any answer >1 day, asked a follow-up question about the weekly frequency of the consumption.

Exposure variables

For the collection of other household construction and characteristics and of the wealth index of the households, standard questions used by Measure Demographic and Health Surveys (DHS) for Demographic Health Surveys were followed; data about household construction and asset ownership was collected, and principal component analysis was conducted to stratify households according to relative wealth [16]. Individual data about sex, age, education, occupational status, physical activity, and food intake was collected from the elderly, and data about household characteristics, occupation, and education was collected from the head of the household. The study collected data on the consumption of food groups during the day prior to the interview and calculated an individual dietary diversity score (DDS). A diet was considered adequately diversified if it included the consumption of five or more food groups out of 10 food groups, using methods typically applied to reproductive-age women. Given the absence of a specific cutoff for the elderly population, this cutoff was adopted to measure the DDS of elderly people [17]. Respondents were divided into three categories (60-64 years, 65-69 years, and 70+ years). Employments were divided into three categories according to the involvement of work. Anthropometric measurements were taken following the Food and Nutrition Technical Assistance III Project (FANTA) anthropometry manual [11], height was measured using a locally made stadiometer, and weight was measured using the TANITA, model UM-070 weighing scale [11]. The body mass index (BMI) of elderly >60 years was calculated and categorized using the Asian cutoff points, such as underweight (<18.5 kg/m^2^), normal (≥18.5 to <23 kg/m^2^), overweight (≥23 to <27.5 kg/m^2^), and obese (≥27.5 kg/m^2^) [18].

Statistical analysis

To address disparities in SCFS, SS, and SSB intake, we stratified our analyses by gender. Weighted analysis revealed outcome variable prevalence and risk factors. Unweighted participant distribution was also presented. Descriptive sociodemographic analysis was conducted for elderly males and females. Linear regression models explored mean intake differences of SCFS, SS, and SSB by exposure categories, showing mean differences, 95% CIs, and p-values for crude and adjusted analysis. Final multivariable regression variables were selected based on bivariate analysis, considering exposure variables with a p-value of ≤0.2 as candidates for inclusion in the final model [19]. Crude and adjusted mean changes with their 95% CIs were calculated. A variance inflation factor (VIF) of >5 was set as a potential problem, although we did not find VIF>5, which showed an absence of multicollinearity among exposure variables.

Ethical issues

Before commencing the research, ethical clearance (reference: 2018-020-IR) was obtained from the Institutional Review Board of BRAC James P Grant School of Public Health, BRAC University, Dhaka, Bangladesh. Community consent was acquired through informative sessions led by local leaders, and written consent, ensuring anonymity and confidentiality, was obtained prior to data collection. Parental consent was sought for participants under 18. The study's planning, implementation, and dissemination were conducted without involvement with patients or the broader public.

## Results

Table 1 shows the social and demographic status of Bangladeshi elderly people. The total population was subdivided into three age groups where 2,081 (43.2%) of the total population belonged to the 60-64 years age group. Table 1 also presented the educational attainment of the elderly, with 979 (20.3%), 590 (12.2%), and 234 (4.9%) falling into the primary, secondary, and above secondary groups, respectively. However, 3,014 (62.6%) of the population received none; almost half of the males (1,214 (48.9%)) and a significant majority of females (1,800 (77.1%)) fall within the no-education category. Most of the study population was of the Muslim community (4,075 (84.6%)). Moreover, 2,277 (91.7%) of males and 584 (25%) of females were currently married at the time of the interview. Table 1 also illustrates the proportions of underweight and overweight populations at 1,100 (23.9%) and 1,431 (31.1%), respectively, and also indicates that 2,077 (45.1%) of elderly males and females were normal in BMI. More than half of the participants (2,844 (59%)) did not watch television, and 1,989 (41.3%) of the total elderly consumed an inadequately diversified diet.

**Table 1:** Sociodemographic characteristics of the elderly male and female Others^$^: Never married, separated, widowed, divorced, refused Others^$$^: Hindu, Christian, Buddhist BMI: body mass index

	Overall (n=4,817)		Male (n=2,482)		Female (n=2,335)		p-value
	Number	Percent	Number	Percent	Number	Percent	
Age groups (years)							<0.001
60-64	2,081	43.2	1,101	44.4	980	42	
65-69	1,144	23.7	624	25.1	520	22.3	
≥70	1,592	33	757	30.5	835	35.8	
Place of residence							0.003
Rural	3,463	71.9	1,835	73.9	1,628	69.7	
Non-slum urban	807	16.8	394	15.9	413	17.7	
Slum	547	11.4	253	10.2	294	12.6	
Educational attainment							<0.001
No education (grade 0)	3,014	62.6	1,214	48.9	1,800	77.1	
Primary (grades 1-5)	979	20.3	588	23.7	391	16.7	
Secondary (grades 6-10)	590	12.2	465	18.7	125	5.4	
Above secondary (grades >10)	234	4.9	215	8.7	19	0.8	
Marital status							<0.001
Currently married	2,861	59.4	2,277	91.7	584	25	
Others$	1,956	40.6	205	8.3	1,751	75	
Religion							0.29
Islam	4,075	84.6	2,113	85.1	1,962	84	
Others$$	742	15.4	369	14.9	373	16	
BMI							<0.001
Underweight	1,100	23.9	589	24.4	511	23.2	
Normal	2,077	45.1	1,145	47.5	932	42.4	
Overweight and obese	1,431	31.1	675	28	756	34.4	
Waist circumference							<0.001
Male: <90 cm/female: <80 cm	3,227	68.5	1,887	77.1	1,340	59.1	
Male: ≥90 cm/female: ≥80 cm	1,487	31.5	560	22.9	927	40.9	
Dietary diversity							<0.001
Food groups < 5	1,989	41.3	1,098	44.2	891	38.2	
Food groups ≥ 5	2,828	58.7	1,384	55.8	1,444	61.8	
Television time per day							<0.001
None	2,844	59	1,298	52.3	1,546	66.2	
Up to 60 minutes	1,289	26.8	792	31.9	497	21.3	
>60 minutes	684	14.2	392	15.8	292	12.5	
Household characteristics							
Sex of the household head							<0.001
Female	1,302	27	265	10.7	1,037	44.4	
Male	3,515	73	2,217	89.3	1,298	55.6	
Household wealth quintile							0.015
Poorest	967	20.1	542	21.8	425	18.2	
Poorer	962	20	506	20.4	456	19.5	
Middle	965	20	482	19.4	483	20.7	
Richer	962	20	476	19.2	486	20.8	
Richest	960	19.9	476	19.2	484	20.7	

Table 2 presents the consumption of SCFS, SS, and SSB by elderly males and females during the last seven days preceding the interview. The weighted mean weekly frequency of SCFS consumption among elderly males was higher than among elderly females (male: 1.06±1.89 times versus female: 0.53±1.27 times, p<0.0001). More males (93 (3.19%)) consumed SCFS at least seven times in the previous week, and this proportion was lower among females (27 (0.87%)). Similarly, the weighted mean frequency of SS and SSB consumption in the last seven days were higher among males (4.04±5.80 times and 12.33±12.08 times, respectively, p<0.0001) compared to females (2.66±3.72 times and 5.07±6.84 times, respectively). Regarding SS, 628 (21.67%) males and 371 (13.19%) females consumed at least seven times a week. Strikingly, 1,626 (55.02%) males and 878 (24.34%) females consumed SSB at least seven times in the week preceding the interview.

**Table 2:** Elderly male and female consumption of SCFS, SS, and SSB during the last seven days in Bangladesh ^a^Weighted for study design ^b^SD SCFS: savory crispy or fried snacks, SS: sweet snacks, SSB: sugar-sweetened beverages, SD: standard deviation

Characteristics	Male	Female	Male	Female	Male	Female	Male	Female
	Never		1-3 times		4-6 times		7 or more	
SCFS								
Meana (±SD)b	Male: 1.06±1.89				Female: 0.53±1.27			p<0.000
Prevalence (%)	68.54 (63.01,73.59)	79.92 (74.55,84.39)	22.76 (19.34,26.60)	16.75 (12.96,21.37)	5.50 (3.71,8.08)	2.46 (1.59,3.77)	3.19 (2.23,4.55)	0.87 (0.39,1.93)
SS								
Meana (±SD)b	Male: 4.04±5.80				Female: 2.66±3.72			p<0.000
Prevalence (%)	33.86 (27.75,40.56)	46.06 (39.39,52.88)	31.92 (28.14,35.96)	30.72 (26.48,35.32)	12.55 (9.73,16.04)	10.03 (7.84,12.75)	21.67 (16.88,27.37)	13.19 (8.29,20.33)
SSB								
Meana (±SD)b	Male: 12.33±12.08				Female: 5.07±6.84			p<0.000
Prevalence (%)	30.65 (23.69,38.61)	65.85 (53.28,76.53)	10.00 (8.04,12.37)	7.35 (5.35,10.01)	4.33 (3.31,5.65)	2.45 (1.52,3.95)	55.02 (46.40,63.36)	24.34 (14.97,37.03)

Table 3 displays the association of SCFS consumption during the last seven days with background characteristics among the elderly in Bangladesh. In the multiple linear regression model, after adjusting for potential confounders, the mean frequency of weekly consumption of SCFS was significantly higher among males who belonged to the non-slum urban dwelling (mean difference (95% CI): 0.49 (0.21, 0.77), (p≤0.001)), males watching television for up to 60 minutes daily (mean difference (95% CI): 0.34 (0.08, 0.61), (p≤0.01)), and males watching television for greater than one hour (mean difference (95% CI): 0.41 (0.01, 0.80), (p≤0.05)). Two categories of male education, partial primary education and partial secondary education, were also associated with a mean weekly frequency of increased intake of SCFS (p<0.05).

**Table 3:** Sociodemographic characteristics associated with mean changes in the consumption of SCFS during the last seven days among elderly males and females in Bangladesh ^§^Weighted for study design *p≤0.05 **p≤0.01 ***p≤0.001 Others^$^: Never married, separated, widowed, divorced, refused Others^$$^: Hindu, Christian, Buddhist SCFS: savory crispy or fried snacks, CI: confidence interval, BMI: body mass index

Characteristics	Elderly male			Elderly female		
	Mean frequency of intake (95% CI)§	Unadjusted mean difference (95% CI)	Adjusted mean difference (95% CI)	Mean frequency of intake (95% CI) §	Unadjusted mean difference (95% CI)	Adjusted mean difference (95% CI)
Age groups (years)						
60-64	1.02 (0.79, 1.25)	Reference	Reference	0.43 (0.27, 0.58)	Reference	Reference
65-69	1.00 (0.70, 1.29)	-0.02 (-0.25, 0.21)	-0.01 (-0.22, 0.20)	0.50 (0.29, 0.72)	0.07 (-0.13, 0.27)	-
≥70	0.78 (0.56, 0.99)	-0.24 (-0.44, -0.04)*	-0.15 (-0.36, 0.06)	0.47 (0.31, 0.62)	0.04 (-0.13, 0.21)	-
Place of residence						
Rural	0.92 (0.71, 1.12)	Reference	Reference	0.45 (0.32, 0.59)	Reference	Reference
Non-slum urban	1.35 (1.19, 1.5)	0.43 (0.17, 0.69)**	0.49 (0.21, 0.77)***	0.59 (0.47, 0.72)	0.14 (-0.04, 0.32)	0.07 (-0.16, 0.30)
Slum	1.33 (0.38, 2.28)	0.41 (-0.56, 1.39)	0.43 (-0.50, 1.36)	0.87 (0.46, 1.29)	0.42 (-0.01, 0.85)*	0.44 (-0.02, 0.90)
Educational attainment						
No education (grade 0)	0.78 (0.59, 0.97)	Reference	Reference	0.36 (0.26, 0.46)	Reference	Reference
Primary (grades 1-5)	1.06 (0.77, 1.36)	0.29 (0.05, 0.52)*	0.28 (0.04, 0.52)*	0.89 (0.61, 1.17)	0.53 (0.29, 0.77)***	0.50 (0.27, 0.74)***
Secondary (grades 6-10)	1.06 (0.81, 1.32)	0.29 (0.05, 0.52)*	0.26 (0.01, 0.51)*	0.34 (0.02, 0.66)	-0.02 (-0.31, 0.27)	-0.11 (-0.41, 0.20)
Above secondary (grades >10)	1.14 (0.67, 1.62)	0.37 (-0.07, 0.81)	0.27 (-0.17, 0.71)	1.45 (0.26, 2.65)	1.09 (-0.06, 2.25)	0.91 (-0.23, 2.06)
Marital status						
Others$	1.08 (0.66, 1.5)	Reference	-	0.44 (0.29, 0.59)	Reference	-
Currently married	0.92 (0.71, 1.12)	-0.17 (-0.58, 0.25)	-	0.48 (0.3, 0.66)	0.04 (-0.15, 0.24)	-
Religion						
Others$$	1.05 (0.72, 1.39)	Reference	-	0.50 (0.26, 0.75)	Reference	-
Islam	0.90 (0.68, 1.12)	-0.15 (-0.55, 0.24)	-	0.45 (0.30, 0.59)	-0.05 (-0.33, 0.22)	-
BMI						
Normal	0.94 (0.73, 1.16)	Reference	-	0.48 (0.33, 0.63)	Reference	-
Underweight	0.96 (0.68, 1.24)	0.02 (-0.23, 0.26)	-	0.40 (0.24, 0.56)	-0.07 (-0.27, 0.12)	-
Overweight and obese	0.94 (0.68, 1.2)	-0.01 (-0.19, 0.18)	-	0.50 (0.26, 0.73)	0.02 (-0.18, 0.23)	-
Waist circumference						
Male: <90 cm/female: <80 cm	0.92 (0.70, 1.13)	Reference	-	0.44 (0.33, 0.55)	Reference	-
Male: ≥90 cm/female: ≥80 cm	1.00 (0.71, 1.29)	0.09 (-0.18, 0.35)	-	0.51 (0.28, 0.74)	0.07 (-0.14, 0.27)	-
Dietary diversity						
Food groups < 5	0.90 (0.67, 1.12)	Reference	-	0.40 (0.29, 0.50)	Reference	Reference
Food groups ≥ 5	0.96 (0.72, 1.21)	0.07 (-0.17, 0.31)	-	0.55 (0.35, 0.74)	0.15 (0.02, 0.28)*	0.12 (-0.01, 0.24)
Television time per day						
None	0.74 (0.55, 0.93)	Reference	Reference	0.42 (0.29, 0.55)	Reference	Reference
Up to 60 minutes	1.13 (0.81, 1.44)	0.39 (0.12, 0.65)**	0.34 (0.08, 0.61)**	0.52 (0.32, 0.72)	0.09 (-0.10, 0.29)	-
>60 minutes	1.19 (0.81, 1.56)	0.45 (0.05, 0.85)*	0.41 (0.01, 0.80)*	0.61 (0.26, 0.95)	0.18 (-0.16, 0.53)	-
Household characteristics						
Sex of household head						
Male	0.91 (0.71, 1.11)	Reference	Reference	0.41 (0.3, 0.53)	Reference	Reference
Female	1.08 (0.69, 1.47)	0.16 (-0.19, 0.52)	-	0.55 (0.33, 0.77)	0.14 (-0.07, 0.35)	-
Household wealth quintile						
Poorest	0.83 (0.60, 1.06)	Reference	Reference	0.43 (0.26, 0.59)	Reference	Reference
Poorer	1.00 (0.65, 1.36)	0.17 (-0.15, 0.49)	0.15 (-0.17, 0.48)	0.41 (0.25, 0.58)	-0.01 (-0.22, 0.19)	-0.04 (-0.23, 0.16)
Lower	1.03 (0.76, 1.30)	0.20 (-0.08, 0.49)	0.14 (-0.13, 0.41)	0.33 (0.22, 0.44)	-0.10 (-0.28, 0.08)	-0.10 (-0.28, 0.08)
Richer	0.78 (0.47, 1.10)	-0.05 (-0.38, 0.28)	-0.11 (-0.42, 0.20)	0.52 (0.26, 0.78)	0.09 (-0.15, 0.34)	0.05 (-0.18, 0.28)
Richest	1.00 (0.73, 1.27)	0.17 (-0.05, 0.39)	-0.05 (-0.36, 0.27)	0.66 (0.43, 0.89)	0.23 (-0.02, 0.49)	0.12 (-0.11, 0.35)

Among females after adjusting for potential confounders, the mean frequency of weekly consumption of SCFS was significantly higher among females belonging to the primary (grades 1-5) group (mean difference (95% CI): 0.50 (0.27, 0.74), (p≤0.001)). However, no association between other studied characteristics and consumption of SCFS was identified among females.

The association of SS consumption during the last seven days with background characteristics among elderly males and females in Bangladesh is shown in Table 4. In the multiple linear regression model, after adjusting for potential confounders, the mean frequency of weekly consumption of SS was significantly higher among males residing in slum areas (mean difference (95% CI): 3.81 (0.05,7.57), (p≤0.05)) and among males watching television for more than 60 minutes daily (mean difference (95% CI): 0.95 (0.05, 1.85), (p≤0.05)). The mean weekly frequency of SS intake was higher for males from female-headed households (mean difference (95% CI): 2.78 (0.98, 4.58), (p≤0.01)) and males who belonged to primary (grades 1-5) and secondary (grades 6-10) groups (p≤0.05). The mean weekly frequency of consumption of SS was lower among currently married males (mean difference (95% CI): -2.61 (-3.96, -1.25), (p≤0.0001)).

**Table 4:** Sociodemographic characteristics associated with mean changes in the consumption of SS during the last seven days among elderly males and females in Bangladesh ^§^Weighted for study design *p≤0.05 **p≤0.01 ***p≤0.001 Others^$^: Never married, separated, widowed, divorced, refused Others^$$^: Hindu, Christian, Buddhist SS: sweet snacks, CI: confidence interval, BMI: body mass index

Characteristics	Elderly male			Elderly female		
	Mean frequency of intake (95% CI)§	Unadjusted mean difference (95% CI)	Adjusted mean difference (95% CI)	Mean frequency of intake (95% CI)§	Unadjusted mean difference (95% CI)	Adjusted mean difference (95% CI)
Age groups (years)						
60-64	3.63 (2.72, 4.54)	Reference	Reference	2.36 (1.58, 3.13)	Reference	Reference
65-69	3.46 (2.61, 4.31)	-0.17 (-0.77, 0.42)	-	2.29 (1.41, 3.17)	-0.06 (-0.63, 0.51)	-
≥70	3.28 (2.64, 3.92)	-0.35 (-1.07, 0.37)	-	2.21 (1.52, 2.9)	-0.14 (-0.54, 0.25)	-
Place of residence						
Rural	3.44 (2.73, 4.16)	Reference	Reference	2.29 (1.56, 3.03)	Reference	Reference
Non-slum urban	4.21 (2.85, 5.57)	0.77 (-0.77, 2.31)	0.74 (-0.6, 2.08)	2.3 (1.65, 2.95)	0.01 (-0.97, 0.99)	-
Slum	7.58 (4.4, 10.76)	4.14 (0.88, 7.40)*	3.81 (0.05, 7.57)*	2.96 (2.04, 3.89)	0.67 (-0.51, 1.85)	-
Educational attainment						
No education (grade 0)	3.07 (2.36, 3.79)	Reference	Reference	2.09 (1.47, 2.72)	Reference	Reference
Primary (grades 1-5)	3.75 (2.94, 4.56)	0.68 (0.05, 1.30)*	0.63 (0.002, 1.26)*	2.79 (1.54, 4.05)	0.7 (-0.17, 1.57)	0.44 (-0.34, 1.21)
Secondary (grades 6-10)	4.01 (3.04, 4.99)	0.94 (0.04, 1.85)	0.91 (0.18, 1.64)*	3.79 (1.97, 5.6)	1.69 (0.08, 3.31)*	1.21 (-0.38, 2.8)
Above secondary (grades >10)	3.89 (2.29, 5.5)	0.82 (-0.69, 2.34)	0.66 (-0.62, 1.93)	7.35 (4.25, 10.45)	5.26 (2.29, 8.23)***	4.20 (1.58, 6.83)**
Marital status						
Others$	6.00 (4.11, 7.89)	Reference	Reference	2.41 (1.71, 3.1)	Reference	Reference
Currently married	3.31 (2.67, 3.94)	-2.69 (-4.17, -1.22)***	-2.61 (-3.96, -1.25)***	2.12 (1.28, 2.96)	-0.29 (-0.75, 0.17)	-
Religion						
Others$$	4.17 (2.21, 6.13)	Reference	Reference	2.92 (1.32, 4.52)	Reference	Reference
Islam	3.33 (2.67, 3.98)	-0.84 (-2.76, 1.08)	-	2.16 (1.47, 2.85)	-0.76 (-2.29, 0.77)	-
BMI						
Normal	3.41 (2.54, 4.29)	Reference	Reference	2.23 (1.36, 3.1)	Reference	Reference
Underweight	3.64 (2.75, 4.53)	0.23 (-0.55, 1.01)	-	2.24 (1.47, 3.01)	0.01 (-0.59, 0.61)	0.17 (-0.35, 0.68)
Overweight and obese	3.54 (2.8, 4.27)	0.13 (-0.6, 0.85)	-	2.64 (1.81, 3.47)	0.41 (-0.19, 1.02)	0.23 (-0.32, 0.78)
Waist circumference						
Male: <90 cm/female: <80 cm	3.45 (2.7, 4.19)	Reference	Reference	2.15 (1.44, 2.86)	Reference	Reference
Male: ≥90 cm/female: ≥80 cm	3.62 (2.84, 4.4)	0.17 (-0.48, 0.83)	-	2.59 (1.64, 3.54)	0.44 (-0.26, 1.15)	-
Dietary diversity						
Food groups <5	3.39 (2.62, 4.17)	Reference	Reference	2.37 (1.61, 3.12)	Reference	Reference
Food groups ≥5	3.56 (2.84, 4.27)	0.16 (-0.33, 0.65)	-	2.18 (1.42, 2.94)	-0.18 (-0.68, 0.31)	-
Television time per day						
None	3.17 (2.53, 3.8)	Reference	Reference	2.13 (1.4, 2.86)	Reference	Reference
Up to 60 minutes	3.67 (2.74, 4.6)	0.51 (-0.08, 1.09)	0.43 (-0.07, 0.94)	2.57 (1.75, 3.39)	0.44 (-0.24, 1.11)	-
>60 minutes	4.21 (3.04, 5.39)	1.05 (-0.09, 2.19)	0.95 (0.05, 1.85)*	3.03 (1.37, 4.68)	0.90 (-0.70, 2.49)	-
Household characteristics						
Sex of household head						
Male	3.23 (2.65, 3.81)	Reference	Reference	2.02 (1.38, 2.66)	Reference	Reference
Female	6.25 (4.03, 8.46)	3.02 (1.02, 5.01)**	2.78 (0.98, 4.58)**	2.88 (1.95, 3.81)	0.86 (0.22, 1.49)**	0.80 (0.26, 1.35)**
Household wealth quintile						
Poorest	3.33 (2.43, 4.23)	Reference	Reference	2.02 (1.33, 2.71)	Reference	Reference
Poorer	3.96 (2.91, 5.00)	0.63 (0.07, 1.19)*	0.62 (-0.01, 1.26)	2.27 (1.34, 3.19)	0.25 (-0.25, 0.75)	0.20 (-0.32, 0.72)
Lower	3.17 (2.45, 3.9)	-0.15 (-0.96, 0.65)	-0.27 (-1.11, 0.58)	1.93 (1.42, 2.44)	-0.09 (-0.63, 0.45)	-0.27 (-0.80, 0.26)
Richer	2.82 (2.26, 3.38)	-0.51 (-1.48, 0.47)	-0.58 (-1.48, 0.32)	2.04 (1.13, 2.95)	0.02 (-0.59, 0.63)	-0.08 (-0.74, 0.58)
Richest	4.15 (2.79, 5.52)	0.83 (-0.29, 1.94)	0.20 (-0.74, 1.14)	3.59 (2.43, 4.76)	1.58 (0.71, 2.45)***	1.43 (0.64, 2.22)***

Females with 10^th^ grade or higher level of education (mean difference (95% CI): 4.20 (1.58, 6.83), (p≤0.01)), females from female-headed households (mean difference (95% CI): 0.80 (0.26, 1.35), (p≤0.01)), and females from the richest wealth quintile (mean difference (95% CI): 1.43 (0.64, 2.22), (p≤0.001)) were associated with higher weekly frequency of intake of SS.

Table 5 displays the association of SSB consumption during the last week with their background characteristics among elderly males and females in Bangladesh. After adjustment for potential confounders, the mean weekly frequency of consumption of SSB was significantly lower among males of the +70 age group (mean difference (95% CI): -2.02 (-3.46, -0.58), (p≤0.01)) and among overweight and obese males (mean difference (95% CI): -2.05 (-3.76, -0.34) (p≤0.05)). The mean weekly frequency of SSB consumption among males residing in both non-slum and slum urban areas, those from female-headed households, and those who watched television for 60 minutes daily or more was significantly higher.

**Table 5:** Sociodemographic characteristics associated with mean changes in the consumption of SSB during the last seven days among elderly males and females in Bangladesh ^§^Weighted for study design *p≤0.05 **p≤0.01 ***p≤0.001 Others^$^: Never married, separated, widowed, divorced, refused Others^$$^: Hindu, Christian, Buddhist SSB: sugar-sweetened beverages, CI: confidence interval, BMI: body mass index

Characteristics	Elderly male			Elderly female		
	Mean frequency of intake (95% CI)§	Unadjusted mean difference (95% CI)	Adjusted mean difference (95% CI)	Mean frequency of intake (95% CI)§	Unadjusted mean difference (95% CI)	Adjusted mean difference (95% CI)
Age groups (years)						
60-64	11.03 (8.95, 13.12)	Reference	Reference	3.55 (1.77, 5.34)	Reference	Reference
65-69	10.26 (8.04, 12.49)	-0.77 (-2.46, 0.92)	-0.45 (-2.00, 1.10)	3.49 (1.49, 5.50)	-0.06 (-1.06, 0.93)	-
≥70	8.00 (6.34, 9.67)	-3.03 (-4.48, -1.58)***	-2.02 (-3.46, -0.58)**	3.20 (1.58, 4.82)	-0.36 (-1.19, 0.48)	-
Place of residence						
Rural	9.61 (7.78, 11.45)	Reference	Reference	3.32 (1.6, 5.04)	Reference	Reference
Non-slum urban	16.66 (14.35, 18.97)	7.05 (4.09, 10.00)***	6.68 (2.99, 10.38)***	9.2 (6.39, 12.01)	5.88 (2.59, 9.18)***	5.55 (1.65, 9.45)**
Slum	22.02 (15.35, 28.69)	12.41 (5.49, 19.32)***	11.38 (3.17, 19.58)**	7.79 (5.75, 9.82)	4.47 (1.8, 7.14)***	3.64 (0.81, 6.46)**
Educational attainment						
No education (grade 0)	9.13 (6.98, 11.29)	Reference	Reference	3.13 (1.55, 4.71)	Reference	Reference
Primary (grades 1-5)	9.61 (7.25, 11.98)	0.48 (-1.92, 2.88)	0.48 (-1.72, 2.68)	4.2 (1.76, 6.65)	1.07 (-0.37, 2.52)	0.21 (-0.62, 1.04)
Secondary (grades 6-10)	10.79 (8.53, 13.04)	1.65 (-0.45, 3.76)	0.98 (-1, 2.96)	4.99 (2.26, 7.71)	1.86 (-0.53, 4.24)	0.16 (-2.23, 2.54)
Above secondary (grades >10)	12.5 (8.82, 16.17)	3.36 (-0.74, 7.47)	1.67 (-3.1, 6.43)	13.26 (12.02, 14.5)	10.13 (8.18, 12.08)***	6.38 (2.8, 9.96)***
Marital status						
Others$	10.36 (7.32, 13.41)	Reference	Reference	3.72 (1.97, 5.47)	Reference	Reference
Currently married	9.72 (7.92, 11.52)	-0.64 (-2.98, 1.69)	-	2.96 (1.26, 4.65)	-0.76 (-1.51, -0.02)*	0.01 (-0.8, 0.82)
Religion						
Others$$	11.33 (7.8, 14.86)	Reference	Reference	5.81 (1.91, 9.72)	Reference	Reference
Muslim	9.46 (7.56, 11.35)	-1.88 (-5.59, 1.84)	-	2.92 (1.33, 4.51)	-2.89 (-6.66, 0.88)	-2.94 (-5.99, 0.12)
BMI						
Normal	10.26 (8.24, 12.27)	Reference	Reference	3.7 (1.64, 5.77)	Reference	Reference
Underweight	9.42 (7.41, 11.43)	-0.84 (-2.11, 0.44)	0.04 (-1.15, 1.23)	3 (1.5, 4.5)	-0.7 (-1.75, 0.35)	-0.21 (-0.9, 0.49)
Overweight and obese	10.26 (8.15, 12.37)	0.004 (-1.76, 1.77)	-2.05 (-3.76, -0.34)*	3.88 (1.97, 5.78)	0.17 (-1.06, 1.41)	0.11 (-0.93, 1.14)
Waist circumference						
Male: <90 cm/female: <80 cm	9.52 (7.54, 11.49)	Reference	Reference	3.28 (1.59, 4.97)	Reference	Reference
Male: ≥90 cm/female: ≥80 cm	11.13 (9.05, 13.21)	1.62 (-0.41, 3.64)	2.12 (-0.17, 4.41)	3.8 (1.86, 5.73)	0.51 (-0.49, 1.52)	-
Dietary diversity						
Food groups < 5	9.6 (7.66, 11.54)	Reference	Reference	3.43 (1.76, 5.11)	Reference	Reference
Food groups ≥ 5	9.97 (7.97, 11.96)	0.37 (-1.11, 1.85)	-	3.39 (1.55, 5.24)	-0.04 (-0.92, 0.84)	-
Television time per day						
None	7.95 (6.26, 9.63)	Reference	Reference	2.81 (1.16, 4.46)	Reference	Reference
Up to 60 minutes	11.44 (9.22, 13.66)	3.49 (2.16, 4.82)***	2.82 (1.57, 4.06)***	4.29 (2.54, 6.04)	1.48 (0.02, 2.94)*	0.79 (-0.64, 2.21)
>60 minutes	13.14 (10.54, 15.74)	5.19 (2.87, 7.51)***	4.01 (1.62, 6.4)***	6.6 (3.17, 10.03)	3.79 (0.9, 6.68)**	2.23 (0.52, 3.95)**
Household characteristics						
Sex of household head						
Male	9.41 (7.7, 11.11)	Reference	Reference	2.34 (1.13, 3.54)	Reference	Reference
Female	13.96 (10.06, 17.86)	4.55 (1.19, 7.92)**	4.78 (2.07, 7.49)***	5.72 (3.24, 8.21)	3.39 (1.66, 5.11)***	3.42 (1.86, 4.99)***
Household wealth quintile						
Poorest	9.65 (7.53, 11.77)	Reference	Reference	2.94 (1.32, 4.56)	Reference	Reference
Poorer	9.74 (7.49, 11.99)	0.09 (-1.29, 1.46)	-0.16 (-1.42, 1.1)	3.77 (1.18, 6.36)	0.83 (-0.77, 2.42)	0.65 (-0.61, 1.91)
Lower	9.73 (7.38, 12.09)	0.08 (-2.00, 2.16)	-0.37 (-2.41, 1.67)	2.53 (1.3, 3.77)	-0.41 (-1.85, 1.03)	-0.87 (-2.27, 0.53)
Richer	8.22 (6.41, 10.03)	-1.43 (-3.37, 0.51)	-1.7 (-3.63, 0.23)	3.06 (1.43, 4.69)	0.12 (-0.81, 1.05)	0.01 (-0.89, 0.91)
Richest	12.14 (9.66, 14.62)	2.48 (0.41, 4.56)*	0.78 (-1.5, 3.06)	5.24 (2.97, 7.52)	2.30 (0.52, 4.09)**	1.29 (-0.26, 2.85)

After adjustment for potential confounders, females residing in the non-slum urban and slum area had significantly higher levels of consumption of SSB (mean difference (95% CI): 5.55 (1.65, 9.45), (p≤0.01)) and (mean difference (95% CI): 3.64 (0.81, 6.46), (p≤0.01)), respectively. Elderly females having the highest level of education (above 10^th^ grade) (mean difference (95% CI): 6.38 (2.8, 9.96), (p≤0.001)), viewing more than 60 minutes of television daily (mean difference (95% CI): 2.23 (0.52, 3.95), (p≤0.01)), and belonging to a female-headed household (mean difference (95% CI): 3.42 (1.86, 4.99), (p≤0.001)) also had a higher weekly frequency of intake of SSB.

## Discussion

Using data from a nationally representative sample, we showed that SCFS, SS, and SSB consumption is high among Bangladeshi elderly males and females. However, only a fraction of the elderly did not consume these foods and drinks within the week preceding the interview. Overall, the weekly frequency of intake of SCFS, SS, and SSB was higher among elderly males than among elderly females. We also explored the association of several sociodemographic variables with the consumption of SCFS, SS, and SSB and reported the associations between the consumption of these unhealthy snacks and drinks and sociodemographic characteristics. Urban dwelling, higher level of educational attainment, television viewing, and dwelling in a female-headed household were associated with a higher frequency of consumption of unhealthy foods and drinks.

Factors associated with intake of SCFS, SS, and SSB

The elderly from urban areas consumed more unhealthy foods and drinks; both elderly males and females from slum and non-slum urban areas consumed more SSB than their rural counterparts. Intake of SCFS was high among elderly males from non-slum urban areas, and intake of SS was higher among elderly males from slum areas than elderly from rural areas. More females in urban areas work outside the home, so members from such households may rely more on processed foods and drinks [20]. A Bangladeshi cross-sectional study analyzing food consumption patterns, using nationally representative data, similarly found that the mean expenditure for foods consumed outside of the home was higher in urban areas across all income groups compared to rural households, thereby supporting our findings [21].

A higher level of television viewing (more than 60 minutes) was associated with increased frequency of consumption of SCFC, SS, and SSB among elderly males. Many studies reported a positive association between the consumption of unhealthy foods and beverages with television viewing among children and adolescents [22]. Relatively less research has been conducted to explore the association between television viewing and food intake among the elderly. Higher intake of unhealthy foods (salty snacks and sweet foods) and beverages was reported among adults and the elderly with higher television exposure in the USA [23]. Among elderly females, television viewing was not associated with intake of SCFC and SS, and only SSB intake was associated with more than one hour of television viewing. The reasons for such gender differences are not clear, but it may be due to their preference in the selection of the types of television programs they enjoy. It was reported from India that elderly males preferred to spend more time getting information through watching the news, while elderly females devote more time to enjoying film and television serials [24]. In future studies, it will be important to study how different programs and food advertisements influence the diet of the elderly. Moreover, during older age, due to various factors such as loneliness, decline in physical ability, and retirement from a job, older adults spend time viewing television [24]. It is assumed that adults and the elderly have better discriminating power to recognize false claims of television commercials and make rational choices, although it was reported that the ability to reasonably analyze information is compromised during old age [23]; as a result, they could be influenced by the aggressive advertisement strategies of the processed food industry.

In our analysis, we found that the educational attainment of the respondents was not associated with the consumption of unhealthy snacks or drinks among elderly males. However, a higher level of education (more than 10^th^ grade) was associated with increased frequency of consumption of both SS and SSB among elderly females. In a systematic review, a higher level of ultra-processed food intake was observed among adults and elderly having high levels of educational attainment [6]. Higher levels of income, involvement with professional and other activities, and longing for more food varieties of more educated females may be associated with higher levels of SS and SSB intake among elderly females, but this hypothesis needs to be explored in future studies.

The elderly of both sexes from female-headed households consumed more SS and SSB. A possible explanation is that the females from such households may have to devote more time to their household and income-earning activities, get less time for the preparation of meals, and rely more on purchased or easy-to-prepare snacks and beverages. It was reported earlier that in Bangladeshi households where female members work in the non-farm sector, consumption of foods prepared away from home was higher [25].

Overweight or obese elderly males consumed SSB less frequently, but nutritional status was not associated with the consumption of these foods and drinks among elderly females. Frequent intake of unhealthy snacks and SSB is reported to be associated with weight gain [26]. So, the lack of association between intake of SCFS, SS, or SSN and overweight or obesity among males or even lower intake of SSB among females is surprising. This could be due to reverse causation, as overweight or obese elderly males restricted their intake of unhealthy foods and SSB [27].

Females from the richest households consumed more SSB. Such findings indicated that SSB may be accepted as a non-harmful drink, or ownership of more assets is associated with regular consumption of SSB, underscoring the importance of nutrition education on the probable adverse health effects of SSB.

Age was not associated with intake of SCFS and SS among elderly males and females, although most senior elderly males (70 years or more) consumed lower levels of SSB, but at this age, intake of all foods and drinks probably decreases. The dietary diversity of the elderly was not associated with the intake of these foods and drinks.

To our knowledge, this is the first attempt in Bangladesh to report the consumption of SFFS, SS, and SSB among older adults and their sociodemographic determinants using data collected from the whole country. In our analysis, we revealed that SCFS, SS, and SSB consumption is high among the elderly. Due to loss of appetite and reduced ability to prepare food, older adults rely more on unhealthy foods such as salty snacks [23]. However, most of the SFFS, SS, and SSB, especially those available in the Bangladeshi markets, are unhealthy [7]. It is important to reduce their consumption and encourage consumption of healthier snacks and beverages for better health.

Strengths and limitations

Data about individual food items within the broad categories of SCFS, SS, and SSB were not collected. We did not collect data about the sources of these foods and drinks, i.e., whether these were prepared at home or purchased from the market, or about the quantity of these foods or drinks consumed by the elderly. The strength of the study is that we collected data from the whole country and covered rural, slum, and non-slum areas from a large number of elderly males and females. We also presented the analysis by a large number of sociodemographic characteristics. This makes our findings more generalizable for Bangladesh.

## Conclusions

Sociodemographic factors such as increased television viewing and urban living are linked to higher consumption of these snacks and beverages, which has important policy implications. Older adults, facing loneliness and reduced physical ability in aging, spend more time watching television. While they may have better discernment, they can still be influenced by aggressive advertising from the processed food industry. This underscores the need for restriction of advertisements for unhealthy foods. Rapid urbanization is making unhealthy processed foods and beverages more and more accessible, but it is important to promote healthier snacks and discourage the marketing of unhealthy ones through introduction of taxes and restrictions for aggressive promotional measures. Promoting nutrition education for the elderly and their caregivers also warranted further attention from policymakers for improving the health and well-being of the elderly. We recommend future studies to identify the most commonly consumed unhealthy foods and beverages, collect data about the sources of these foods, and determine the amount of such foods and drinks consumed by the elderly.
